# Syphilis: Re-emergence of an old foe

**DOI:** 10.15698/mic2016.09.523

**Published:** 2016-06-27

**Authors:** Lola V. Stamm

**Affiliations:** 1 Department of Epidemiology, Gillings School of Global Public Health, University of North Carolina at Chapel Hill, Chapel Hill, NC 27599-7435.

**Keywords:** Syphilis, Treponema pallidum, spirochete, macrolide resistance, re-emerging infectious disease, sexually transmitted infection

## Abstract

Syphilis is caused by infection with *Treponema pallidum* subsp. *pallidum*, a not-yet-cultivable spiral-shaped bacterium that is usually transmitted by sexual contact with an infected partner or by an infected pregnant woman to her fetus. There is no vaccine to prevent syphilis. Diagnosis and treatment of infected individuals and their contacts is key to syphilis control programs that also include sex education and promotion of condom use to prevent infection. Untreated syphilis can progress through four stages: primary (chancre, regional lymphadenopathy), secondary (disseminated skin eruptions, generalized lymphadenopathy), latent (decreased re-occurrence of secondary stage manifestations, absence of symptoms), and tertiary (gummas, cardiovascular syphilis and late neurological symptoms). The primary and secondary stages are the most infectious. WHO estimates that each year 11 million new cases of syphilis occur globally among adults aged 15-49 years. Syphilis has re-emerged in several regions including North America, Western Europe, China and Australia. Host-associated factors that drive the re-emergence and spread of syphilis include high-risk sexual activity, migration and travel, and economic and social changes that limit access to health care. Early, uncomplicated syphilis is curable with a single intramuscular injection of benzathine penicillin G (BPG), the first line drug for all stages of syphilis. Emergence of macrolide-resistant *T. pallidum* has essentially precluded the empirical use of azithromycin as a second-line drug for treatment of syphilis. Virulence attributes of *T. pallidum* are poorly understood. Genomic and proteomic studies have provided some new information concerning how this spirochete may evade host defense mechanisms to persist for long periods in the host.

## INTRODUCTION

Syphilis is one of seven curable, sexually transmitted infections (STIs) that is caused by a bacterial pathogen. The World Health Organization (WHO) estimates that each year 11 million new cases of syphilis occur globally among adults aged 15-49 years 1. Syphilis remains an important public health problem in many low-income countries where it is endemic and it has re-emerged in several high-income countries, particularly in high-risk groups such as men who have sex with men (MSM) 2,3,4. Cases of syphilis in MSM are a major concern because the lesions of early syphilis increase the risk of acquiring and transmitting infection with the human immunodeficiency virus (HIV) 5,6. If left untreated, syphilis can cause irreversible damage to the cardiovascular and central nervous systems, resulting in morbidity and possibly mortality 7. Furthermore, untreated early syphilis in pregnant women results in perinatal death in up to 40% of cases and can cause physical deformity and neurological complications in children who survive 8,9. Control of syphilis is mainly dependent on the timely diagnosis and prompt treatment of infected individuals and their contacts with benzathine penicillin G (BPG), the first-line drug for all stages of syphilis 10.

## ETIOLOGY OF SYPHILIS

The etiological agent of syphilis, *Treponema pallidum* subsp. *pallidum* (*T. pallidum*), was identified by Schaudinn and Hoffmann in 1905 11. *T. pallidum* is an environmentally fragile, microaerophilic, spiral-shaped, motile bacterium. Although it has eluded continuous cultivation *in vitro*, it can be propagated by intratesticular infection of rabbits, the preferred animal model 12. This spirochete is 6-20 µm in length with a diameter of 0.1-0.2 µm. Because of its narrow diameter, *T. pallidum* cannot be visualized by bright-field microscopy. However, it can be visualized with special stains (e.g., silver stain) or by phase-contrast or dark-field microscopy. The latter is an important tool for detection of *T. pallidum* in genital or cutaneous lesions of patients with early syphilis 12.

The cellular architecture of *T. pallidum* is similar to that of Gram-negative bacteria 2,6,12. It has an outer membrane (OM), a thin peptidoglycan cell wall and an inner membrane. However, unlike Gram-negative bacteria, the *T. pallidum* flagella are located in the periplasm, and the OM, which is devoid of lipopolysaccharide (LPS), is more susceptible to disruption by mild physical manipulation and chemical treatments. As demonstrated by freeze-fracture electron microscopy, the *T. pallidum* OM contains only a few integral membrane proteins 2,6,12. Because some of the treponemal rare OM proteins (TROMPs) are exposed on the cell surface, they are likely to be important during infection and are potential vaccine targets. However, the fragility of the *T. pallidum* OM has hindered definitive identification of the TROMPs.

The complete genome sequence of *T. pallidum* Nichols, the type strain, which was determined in 1998, has provided a wealth of information concerning the metabolism and physiology of this spirochete 13. The Nichols genome and that of more recently sequenced strains (e.g., SS14, Chicago, Dal-1, Mexico A) is about 1.14 Mbp, encodes about 1,000 proteins, and lacks genetic elements (e.g., plasmids, bacteriophage, transposons) that are commonly associated with horizontal gene transfer 13,14. The small size of the *T. pallidum* genome is consistent with its limited biosynthetic capabilities, resulting in a long generation time (i.e., 30-33 hours *in vivo*), sensitivity to various environmental conditions (e.g., oxygen, temperature, pH), and dependency on the host for multiple nutrients. *T. pallidum* possesses a single, multigene family encoding 12 paralogous proteins, designated the *T. pallidum* repeat proteins (TprA-TprL). The function and location of these proteins has been a matter of some debate. However, the genetic sequences encoding the TprE, G, and J proteins are, for the most part, sufficiently stable within a strain, but variable among strains, to make them useful for molecular typing when combined with other genomic targets (e.g., *arp* and *tp0548*) 15,16,17. The TprK protein is the most variable of the Tpr proteins with several variants typically observed within a strain as a result of a gene conversion mechanism 6,18,19. Antigenic variation of the TprK protein during infection may promote chronic infection via immune evasion 6,20.

## EPIDEMIOLOGY OF RE-EMERGENT SYPHILIS - U.S. AND CHINA

The U.S. rate of primary and secondary syphilis, the earliest and most infectious stage, declined almost 90% during 1990-2000 21. The low rate of syphilis and the concentration of the majority of cases in a small number of geographic areas prompted the Centers for Disease Control (CDC) to launch a plan in 1999 to eliminate syphilis from the U.S. In 2000, the rate of primary and secondary syphilis was the lowest since reporting began in 1941 (2.1 cases per 100,000 population) 4,21. However, the rate of primary and secondary syphilis increased annually during 2001-2009, decreased slightly in 2010, remained unchanged in 2011, and then increased 22% during 2011- 2013 to 5.3 cases per 100,000 population 4,21. In 2013, men accounted for 91.1% of all primary and secondary syphilis cases with the highest rates among non-Hispanic black men aged 20-29 years 21. MSM accounted for 75% of all primary and secondary syphilis cases. Based on available data (i.e., from 30 states and the District of Columbia), HIV co-infection was present in almost 52% of MSM with syphilis 21. While the majority of primary and secondary syphilis cases occurred in MSM, transmission of syphilis in men who have sex with women continued in some localities and led to an increase in cases of congenital syphilis in pregnant women from 8.4 cases per 100,000 live births in 2012 to 8.7 cases per 100,000 live births in 2013 21. The U.S. South continued to comprise the largest proportion of cases of primary and secondary syphilis in 2013, although the rate in the West exceeded the rate in the South. The total number of syphilis cases (all stages) reported to the CDC increased 13% during 2012-2013 to 56, 471 cases for a rate of 18 cases per 100,000 population 21.

The most dramatic change in reported cases of syphilis in recent decades occurred in China 22,23,24,25. Although syphilis was nearly eliminated in China during the early 1960s, cases began to rise in the 1980s. From 1991 to 2005, syphilis cases increased almost 16-fold to 16% of the total cases of STIs. By 2008, syphilis cases had more than doubled, accounting for almost 38% of the total cases of STIs. In 2013, 444,952 cases of syphilis were reported with a rate of almost 33 cases per 100,000 population. The syphilis epidemic in China is largely driven by economic and social factors, including migration and evolving sexual mores 22,23,24,25. MSM, female sex workers, migrants and drug users are the groups at highest risk for syphilis and for co-infection with HIV 22,23,24,25. Rapid economic development in China has led to an unprecedented number of migrants moving from less developed rural communities to more affluent urban centers. This trend is expected to continue over the next 20 years. Many of the migrants are younger, less educated males who work in low-wage industries or service sectors. Migrants typically lack adequate health insurance, do not use medical facilities due in part to economic reasons, and cannot access social welfare benefits that are available to urban residents. This demographic poses a major public health challenge with respect to STIs because studies suggest that migrants are more likely to engage in STI-associated risk factors (e. g., low condom use, multiple sexual partners, buying and/or selling sex) 22,25.

## CLINICAL MANIFESTATIONS OF UNTREATED ADULT SYPHILIS

Syphilis is a chronic, systemic infection that is typically transmitted by contact with active lesions of a sexual partner (acquired syphilis) 7,26,27. Syphilis can also be transmitted from an infected pregnant woman to her fetus or neonate (congenital syphilis). This STI is characterized by periods of active, clinical disease that are interrupted by periods of latency. Based on clinical findings, untreated adult syphilis can progress through four overlapping stages (i.e., primary, secondary, latent, and tertiary). The primary and secondary stages are the most infectious. Syphilis generally follows a similar course in individuals with and without HIV infection 6,7. (For pictures of the stages of syphilis, see reference 7.)

Primary syphilis occurs following an incubation period of about 2-6 weeks 7,26,27. This stage is characterized by the presence of a painless, indurated ulcer (i.e., chancre) at the site of inoculation (typically the genital, anal or oral mucosa) and by moderate regional lymphadenopathy. In a few weeks, the chancre heals spontaneously due to phagocytosis of the treponemes by activated macrophages, which is enhanced by opsonizing antibodies 6,27. Although most of the treponemes are killed, some escape, possibly due to antigenic variation of the TROMPs, and disseminate to multiple organs via blood and lymph to cause chronic infection.

Secondary syphilis results from widespread multiplication of disseminated *T. pallidum* despite high levels of anti-treponemal antibodies 7,26,27. This stage, which occurs simultaneously with, or up to six months after, the healing of the chancre, is typically characterized by malaise, headache, low-grade fever, generalized lymphadenopathy, a localized or generalized skin rash with lesions on the palms and soles, mucous patches in the oral cavity or genital tract, wart-like lesions (condylomata lata), and patchy hair loss. Secondary syphilis can last for weeks or months. Relapses occur in about one-quarter of untreated patients.

Latent syphilis is the period from the disappearance of the secondary stage manifestations to the occurrence of the tertiary (late) stage manifestations 7,26,27. Latent syphilis is arbitrarily divided into early latent (i.e., within one year of infection) and late latent (i.e., after one year of infection). Clinical symptoms are not apparent during latent syphilis, but serologic tests for detection of antibodies to *T. pallidum* remain positive, indicating that treponemes are still present in the lymph nodes and spleen. However, the absence of exposed mucosal lesions usually precludes venereal transmission of syphilis. About two-thirds of untreated syphilis patients remain in the latent stage for life.

Tertiary (i.e., late) syphilis, which occurred during the pre-antibiotic era in about one-third of untreated syphilis patients, is rare today due to curative antibiotics given for early syphilis or given co-incidentally for unrelated infections 7,26,27. This stage usually presents within several years to a few decades after infection. Tertiary syphilis can affect almost any tissue or organ. Treponemes invade the cardiovascular and central nervous systems, eyes, skin, bones and internal organs. Damage occurs due to the invasive properties and inflammation-provoking cellular components of the treponemes that evoke a delayed-type hypersensitivity response. Manifestations of tertiary syphilis include cardiovascular syphilis, neurosyphilis, and gummatous syphilis. The later involves granulomatous lesions (gummas) that are mostly present in skin and bones, occur singly or multiply, and vary in size from microscopic to large tumor-like masses. Gummas do not usually cause physical incapacity or death, but serious complications can occur if they are present in the brain or heart. Symptoms of tertiary syphilis can include difficulty in movement coordination, paralysis, numbness, blindness, and dementia. Because so few treponemes are present during tertiary syphilis, sexual transmission of syphilis is very unlikely.

## CONGENITAL SYPHILIS

Mother to child transmission (MTCT) of syphilis, commonly known as congenital syphilis, continues to affect pregnant women worldwide and is an important cause of adverse outcomes of pregnancy 27,28,29. The overwhelming proportion of MTCT of syphilis occurs in pregnant women living in low-income countries. Estimates suggest that in 2008, 1.4 million pregnant women had active syphilis, which resulted in 520,000 harmful outcomes including 215,000 stillbirths, 90,000 neonatal deaths, 65,000 pre-term or low birth-weight infants and 150,000 infants with congenital infection 28. *T. pallidum* can be transmitted via the bloodstream of an infected pregnant woman to her developing fetus at any time during pregnancy, although the risk of fetal infection is much higher during untreated, early maternal syphilis than during late stage maternal syphilis. Congenital syphilis is divided into stages with the early manifestations appearing during the first 2 years of age and the late manifestations appearing after 2 years of age 27. Early manifestations are infectious, resembling severe symptoms of adult secondary syphilis, and may include rhinitis, mucocutaneous lesions, osteochondritis of the long bones, hepatosplenomegaly, lymphadenopathy, anemia, jaundice and neurosyphilis. Late manifestations may include interstitial keratitis, eight-nerve deafness, interferences with tooth development, neurosyphilis, perioral fissures (rhagades), damage to long bones (sabre shins) and perforation of the nasal septum (saddle nose). MTCT of syphilis is preventable with early prenatal screening and penicillin treatment of infected pregnant women 10,29. Infants born to women with untreated or inadequately treated syphilis or infants that have physical or laboratory findings that are consistent with syphilis must be treated to prevent progression of the infection 10.

## LABORATORY DIAGNOSIS OF SYPHILIS

Because *T. pallidum* cannot be cultivated *in vitro*, laboratory-based diagnosis of syphilis is usually dependent upon visualization of the spirochete in clinical specimens by dark-field microscopy, which may not be available in low-income countries, and/or reactivity of patient serum or plasma in serological tests 12,17,26,30. Nucleic acid amplification tests, such as the polymerase chain reaction (PCR), have not been used routinely for syphilis diagnosis since a commercial test is not available. However, PCR tests for syphilis can be performed in U.S. clinical laboratories that have developed and verified their own tests in accordance with the Clinical Laboratories Improvement Amendment (CLIA) 31.

Serological tests for syphilis (STS) are traditionally divided into two types: nontreponemal and treponemal 12,17,26,30. These tests measure two distinctly different kinds of antibody reactivities with different kinetics. It is important to note that the currently available STS cannot differentiate venereal syphilis from the endemic treponematoses, which are infections that are caused by the closely related *T. pallidum* subsp. *pertenue* (yaws) and subsp. *endemicum* (bejel) and *T. carateum* (pinta) 12,26. Although PCR combined with genomic sequencing can enable differentiation of the *Treponema pallidum* subspecies, few clinicians have access to these molecular methods 14.

Nontreponemal STS detect IgM and IgG antibodies to lipoidal antigens that are putatively released from damaged host cells 12,26,30. These flocculation tests include the Venereal Disease Research Laboratory (VDRL), rapid plasma reagin (RPR), and toluidine red unheated serum test (TRUST). Nontreponemal tests are widely available, rapid, and inexpensive and can be used quantitatively to monitor the effectiveness of antibiotic treatment (i.e., defined as a 4-fold decline in RPR titer at 6-12 months after treatment). However, the use of nontreponemal STS as screening tests is problematic because they lack sensitivity in primary and tertiary syphilis. Additionally, these tests lack specificity because reactive antibodies can develop in other diseases and medical conditions that are unrelated to syphilis, resulting in biological false positives (BFPs).

Treponemal STS use native or recombinant *T. pallidum* antigens and detect IgM and IgG antibodies to treponemal components 12,26,30. These tests include the *T. pallidum* particle agglutination assay (TPPA), the fluorescent treponemal antibody absorption assay (FTA-ABS), various enzyme-linked immunoassays (EIAs), chemiluminescence immunoassays (CIAs), and rapid point-of-care (POC) immunochromatographic strip assays. In general, treponemal STS are more sensitive and specific than nontreponemal STS for all stages of syphilis and BFPs are less common. However, because treponemal STS cannot distinguish active infection from past or treated infection, they cannot be used to monitor the effectiveness of antibiotic treatment.

U.S. guidelines for serological diagnosis of syphilis recommend the combined use of one nontreponemal STS and one treponemal STS 12,26. The use of only one type of STS is insufficient for diagnosis since each type of STS has its limitations. The traditional algorithm for syphilis diagnosis is based on the use of a nontreponemal STS for screening followed by a treponemal STS for confirmation of a positive screening test. In recent years, several clinical laboratories and blood banks have switched to a “reverse algorithm” in which screening is performed with a treponemal STS followed by a nontreponemal STS when the former test is reactive 17,26,30,32. However, patients with a positive treponemal STS, but a nonreactive nontreponemal STS, could have very early syphilis, long-standing latent syphilis, past treated syphilis or a BFP result. Thus, a good clinical history is essential to distinguish at-risk individuals for whom a positive treponemal STS is likely to be a true positive from individuals whose syphilis was previously treated. For individuals with no history of treated syphilis, a second, but different, treponemal STS of equivalent sensitivity should be performed. A positive second treponemal STS confirms that the patient has or had syphilis. If the second treponemal STS is nonreactive, the clinician may decide that further testing is not needed or that treatment is warranted for individuals at high-risk. There is some concern that use of the reverse algorithm could lead to increased patient follow-ups, unnecessary treatment, and higher medical costs. Several issues must be considered prior to use of this algorithm, including syphilis prevalence and the probability of a patient having syphilis based on individual risk factors.

## TREATMENT, PREVENTION AND CONTROL OF SYPHILIS IN ADULTS

Because there is no vaccine to prevent syphilis, timely diagnosis and treatment of infected individuals and their sexual partners is key to syphilis control programs that also include sex education and promotion of condom use to prevent infection. A major breakthrough for syphilis treatment occurred in 1943 when Mahoney and colleagues reported the use of penicillin to successfully cure patients with primary syphilis 33. Unlike most other bacterial pathogens that quickly developed resistance to penicillin, *T. pallidum* has remained exquisitely sensitive to this antibiotic. The U.S. CDC Sexually Transmitted Diseases (STD) Treatment Guidelines, 2015 recommend BPG as the first-line drug for incubating syphilis and for all stages of syphilis 10. A single intramuscular (IM) injection of 2.4 million units (MU) of BPG is curative for early, uncomplicated syphilis in adults (i.e., primary, secondary, or early latent stage). An IM injection of 2.4 MU of BPG given once weekly for 3 weeks is recommended for late latent syphilis, syphilis of unknown duration and tertiary syphilis. Adults with HIV co-infection should be treated the same as those without HIV infection. Because there are no proven alternatives to penicillin for treatment of pregnant women with syphilis, those who are allergic to penicillin should be desensitized and then treated with penicillin 10,29.

Data to support the use of second-line drugs for syphilis treatment consist mostly of small, uncontrolled, retrospective studies with a few larger, randomized trials 10,26,29. According to the CDC STD treatment guidelines, men and non-pregnant women with early syphilis who are allergic to penicillin may be treated with doxycycline (100 mg orally, twice daily for 14 days) or tetracycline (500 mg orally, four times daily for 14 days) or ceftriaxone (1 g daily either IM or intravenously (IV) for 10-14 days) 10,26. The optimal dose and duration of the latter antibiotic have not been defined. Azithromycin (2 g orally as a single dose) may be used with caution, but only when treatment with penicillin or doxycycline is not feasible. Azithromycin should not be used in MSM, patients with HIV infection, or pregnant women. Careful clinical and serological follow-up of patients receiving any second-line drug is essential because treatment failure can extend the "window of opportunity" for transmission of syphilis 10,17,26,29,34.

## MACROLIDE-RESISTANT *T. PALLIDUM*

Oral azithromycin was shown to be effective for treatment of early syphilis in non-randomized studies and in randomized controlled trials in the U.S, Africa, China, and Madagascar that compared cure rates for this macrolide and penicillin 34,35,36,37,38,39. Azithromycin was used for syphilis treatment in Uganda (mid-1990s), in the U.S. (1999 and 2000) and in Canada (2000). However, during 2002-2003, several cases of clinical failure following azithromycin treatment were observed in syphilis patients in San Francisco, CA 40. Laboratory analysis of samples from these patients showed the presence of an A2058G mutation in the *T. pallidum* 23S rRNA gene. This mutation is identical to the 23S rRNA gene mutation reported earlier by Stamm and Bergen 41 in *T. pallidum* SS14, a clinical isolate from a patient who failed intensive erythromycin treatment, that was shown previously to be highly resistant to erythromycin and cross-resistant to azithromycin 42. Retrospective analysis of samples from syphilis patients in three U.S. cities (i.e., Baltimore, San Francisco, Seattle) and in Vancouver, Canada showed that although the proportion of samples containing *T. pallidum* with the A2058G mutation varied between these sites, it increased significantly over time within the sites 26,34,40,43. Macrolide-resistant *T. pallidum* with the A2058G mutation is now present in the U.S., Canada, Europe, China, and Australia 26,34,43,44,45,46,47,48,49,50,51,52,53. Additionally, in 2009, Matejková *et al*. 44 identified a new mutation, A2059G, in syphilis patients in the Czech Republic that has subsequently been detected in syphilis patients in England, the U.S. and China 48,52,53. Most *T. pallidum* strains containing the A2058G or A2059G mutation can be divided into multiple molecular types indicating that these mutations are not restricted to a single strain type 51,54. The origin of the macrolide-resistant *T. pallidum* strains is unknown. However, Marra *et al*. 54 and others 45,52 reported that these strains were more likely to be found in syphilis patients who had used macrolides previously for treatment of unrelated infections, suggesting that the persistence of low levels of macrolides in a patient’s tissues could have selected for macrolide-resistant *T. pallidum* that arise *de novo*. The geographical prevalence of macrolide-resistant *T. pallidum* varies widely (e.g., 0.7% in Taiwan; nearly 100% in China) 34,45,49,50. In regions where macrolide-resistant *T. pallidum* strains are now highly prevalent, these strains may be able to persist in sexual networks if patients who are infected with macrolide-resistant *T. pallidum* are not treated with curative antibiotics and continue to transmit the infection. Studies in the rabbit model of syphilis indicate that the *T. pallidum* 23S rRNA A2058G mutation is stable in the absence of selective pressure 34,54. Even in regions that currently have low levels of macrolide-resistant *T. pallidum*, the use of macrolides for syphilis treatment cannot continue without adequate surveillance due to the potential introduction of macrolide-resistant strains. Chen *et al*. 55 developed a real-time triplex PCR assay that can rapidly detect the *T. pallidum* A2058G and A2059G mutations. Timely identification of macrolide-resistant *T. pallidum* strains with this assay could be useful for informing physicians as to the treatment of penicillin-allergic patients with early syphilis.

## CONCLUSIONS

Syphilis continues to present challenges to global public health, particularly because it increases the risk of acquiring and transmitting infection with HIV. Nonetheless, syphilis has many features of a disease that could be eliminated 56,57,58. For example, syphilis only affects humans; there is no animal or environmental reservoir. Serodiagnostic tests, though not perfect, are inexpensive and widely available. Early, uncomplicated syphilis is treatable with a single IM injection of BPG, an antibiotic for which *T. pallidum* is unlikely to develop resistance 26,34. Finally, the 2-6 week incubation period of syphilis provides an opportunity to interrupt transmission via prophylactic treatment of sexual contacts. Despite these features, syphilis has resurged in several developed countries including China, which has recently experienced high burdens of several STIs. Host-associated factors that drive the re-emergence and spread of syphilis include high-risk sexual activity, migration and travel, and economic and social changes that limit access to health care 2,22,23,25,59.

In the absence of a vaccine, several obstacles must be addressed to enable the control of infectious syphilis. First, the spread of syphilis is dependent on the average number of new cases that are generated by an infected individual who is most contagious during the primary and secondary stages of the disease 58. The earlier that an infected individual seeks treatment, the sooner the chain of transmission will be broken. However, the typically painless chancre of primary syphilis, which may go undetected if it is present in a less visible body area (e.g., vagina, cervix, anus/rectum), can contribute to persistence and transmission of infection, particularly if individuals do not perceive that they are at risk for syphilis. Additionally, the protean manifestations of secondary syphilis can be misdiagnosed. Sexual health education campaigns targeted to high-risk groups via internet and mobile technologies are critical to raise awareness of syphilis (i.e., risk, symptoms, treatment) and to increase syphilis testing rates 60. Healthcare providers must also be educated to recognize and treat syphilis promptly. Second, availability of simple and accurate POC tests is the key. Newer, dual target POC tests that combine nontreponemal and treponemal antigens, such as the Dual Path Platform (DPP) Syphilis Screen & Confirm test (Chembio Diagnostic Systems Inc., Medford, NY), provide rapid results thereby allowing for same day diagnosis and treatment, which is particularly important for patients who may not return for laboratory-based test results 61,62,63,64. Wider use of the POC tests is critical to expand syphilis screening to high-risk populations and to reduce MTCT of syphilis in low- and middle- income countries. Additionally, syphilis testing provides an opportunity to simultaneously screen for other STIs. Given the high rate of co-infection of syphilis and HIV, POC tests that can detect both infections have been developed and are undergoing further evaluation 65. Third, improved contact tracing of the sexual partners of syphilis patients is crucial to enable prophylactic treatment. This can be complicated, particularly with MSM who use websites and smart phone dating apps to seek anonymous sexual partners 60. However, internet-based strategies to address this issue have been used successfully to locate, test, and treat anonymous partners and to alert MSM to syphilis. Fourth, because of the emergence and increasing prevalence of macrolide-resistant *T. pallidum*, the use of oral azithromycin as a second-line drug for treatment of syphilis is essentially precluded 10,26. Although there is as yet no documented resistance of *T. pallidum* to the tetracyclines, the other main class of second-line drugs, patient compliance is likely to be an issue 26,34,66. Thus, new, single-dose oral antibiotics are needed, particularly in resource-limited settings where IM injections are problematic or for non-pregnant, penicillin-allergic patients. Fifth, the majority of syphilis cases today are in individuals who typically engage in high-risk sexual behavior (e.g., MSM, commercial sex workers, migrants, drug users) and who may have limited access to health care as a result of economic issues and/or social stigmatization. Syphilis testing and treatment need to be widely available, affordable, and destigmatized to enable access by these groups. In the 1980s, changes in sexual behavior were more achievable because of the impact of HIV infection and the lack of an effective treatment. While behavioral change campaigns may not engender as great an effect today, they are still important to encourage consistent use of latex condoms and reduction in partner numbers - actions that benefit the control of syphilis as well as other STIs 59,60. Finally, surveillance must be strengthened to identify new syphilis outbreaks, thereby enabling a rapid response for treatment of infected individuals and their contacts, and to determine which intervention strategies are working and warrant expansion. Although eradication of syphilis may not be possible at this juncture, prior experience has shown that elimination (i.e., regional control) of syphilis is achievable provided that we make optimal use of the tools at hand and do not allow ourselves to again become complacent.
